# Head and body cues guide eye movements and facilitate target search in real-world videos

**DOI:** 10.1167/jov.23.6.5

**Published:** 2023-06-09

**Authors:** Nicole X. Han, Miguel P. Eckstein

**Affiliations:** 1 Department of Psychological and Brain Sciences, Institute for Collaborative Biotechnologies, University of California, Santa Barbara, CA, USA

**Keywords:** gaze cues, attention, eye movements, dynamic gaze

## Abstract

Static gaze cues presented in central vision result in observer shifts of covert attention and eye movements, and benefits in perceptual performance in the detection of simple targets. Less is known about how dynamic gazer behaviors with head and body motion influence search eye movements and performance in perceptual tasks in real-world scenes. Participants searched for a target person (yes/no task, 50% presence), whereas watching videos of one to three gazers looking at a designated person (50% valid gaze cue, looking at the target). To assess the contributions of different body parts, we digitally erase parts of the gazers in the videos to create three different body parts/whole conditions for gazers: floating heads (only head movements), headless bodies (only lower body movements), and the baseline condition with intact head and body. We show that valid dynamic gaze cues guided participants’ eye movements (up to 3 fixations) closer to the target, speeded the time to foveate the target, reduced fixations to the gazers, and improved target detection. The effect of gaze cues in guiding eye movements to the search target was the smallest when the gazer's head was removed from the videos. To assess the inherent information about gaze goal location for each body parts/whole condition, we collected perceptual judgments estimating gaze goals by a separate group of observers with unlimited time. Observers’ perceptual judgments showed larger estimate errors when the gazer's head was removed. This suggests that the reduced eye movement guidance from lower body cueing is related to observers’ difficulty extracting gaze information without the presence of the head. Together, the study extends previous work by evaluating the impact of dynamic gazer behaviors on search with videos of real-world cluttered scenes.

## Introduction

### Gaze information orients overt attention

The gaze direction, head orientation, and body posture of people around us reveal essential information about their internal mental states, intentions, and potential future actions (Azarian, Buzzell, Esser, Dornstauder, & Peterson, 2017; Bayliss, di Pellegrino, & Tipper, 2004a; Emery, 2000; Kleinke, 1986). Daily social interactions involve inferring others’ attention to plan one's future actions. Humans automatically follow with eye movements the gaze direction of others when trying to infer the locus of attention of other individuals. This gaze-following behavior is present in infants as early as 10 months old (Brooks & Meltzoff, 2005). Similar behaviors are also widely observed in nonhumans, such as apes, monkeys, dogs, and goats (Bräuer, Call, & Tomasello, 2005; Brooks & Meltzoff, 2005; Kaminski, Riedel, Call, & Tomasello, 2005; Senju & Csibra, 2008; Shepherd, 2010; Wallis et al., 2015).

It is difficult for humans to ignore others’ eye and head gaze shifts. Studies have shown that centrally presented gaze, head, and body posture induce attention shifts even when the gaze direction is nonpredictive of the target location (Bayliss et al., 2004a; Driver et al., 1999; Friesen & Kingstone, 1998; Friesen, Ristic, & Kingstone, 2004; Hietanen, 1999; Kingstone, Friesen, & Gazzaniga, 2000; McKee, Christie, & Klein, 2007; Ristic, Wright, & Kingstone, 2007). Therefore, most agree to describe gaze shift as an exogenous cue because of its robust effect on shifting attention. However, their temporal development is different. Exogenous attention is defined to be involuntary, transient, and usually triggered by sudden changes in the environment. The effect of exogenous attention typically peaks within 100 ms and quickly dissipates around 150 ms to 200 ms (Cheal & Lyon, 1991; Maylor & Hockey, 1985; Mulckhuyse & Theeuwes, 2010; Müller & Findlay, 1988; Müller & Rabbitt, 1989; Nakayama & Mackeben, 1989; Posner & Cohen, 1984; Shepherd & Müller, 1989; Theeuwes, 1991). However, the time course of covert attention for gaze cues is different. Compared to a typical peripheral exogenous cue (e.g. a flash in the visual periphery), the effect of gaze cue appears as early as 100 ms, persists to 300 to 500 ms from cue onset, and decays afterward (Bayliss et al., 2004a; Friesen & Kingstone, 1998; McKee et al., 2007; Müller & Findlay, 1988; Posner & Cohen, 1984; Shepherd & Müller, 1989; Theeuwes, 1991).

### Gaze as a dynamic behavior

The majority of studies use static images of the eyes, face, or body postures to study the cueing effects on both covert attention (Bayliss, di Pellegrino, & Tipper, 2004b; Driver et al., 1999; Friesen & Kingstone, 1998) and overt attention (Friesen & Kingstone, 2003; Hood, Willen, & Driver, 1998; Mansfield, Farroni, & Johnson, 2003; Ricciardelli, Bricolo, Aglioti, & Chelazzi, 2003), and perceptual performance with simple tasks such as dot detection or letter identification. However, gaze-following is fundamentally a dynamic behavior. Some studies have used simplified dynamics of gaze behaviors, including moving point lights or a single animation of someone's face (Hermens & Walker, 2012; Kuhn & Tipples, 2011; Rutherford & Krysko, 2008; Shi, Weng, He, & Jiang, 2010; Sun, Stein, Liu, Ding, & Nie, 2017; Wang, Yang, Shi, & Jiang, 2014). Recently, a few studies have also started to use realistic videos to study how gaze cues affect attention in natural scenarios (Gregory, 2021; Han & Eckstein, 2022).

### Gaze cueing and visual search

The role of gaze following in more complex tasks, such as visual search in cluttered scenes, has not been studied. Observer's performance during the visual search is degraded by the spatial uncertainty of the target (Bochud, Abbey, & Eckstein, 2004; Burgess & Ghandeharian, 1984; Shimozaki, Schoonveld, & Eckstein, 2012; Swensson & Judy, 1981), distractors that are potentially confused with the target (Nagy, Neriani, & Young, 2005; Palmer, Verghese, & Pavel, 2000), the effect of clutter (Henderson, Chanceaux, & Smith, 2009; Rosenholtz, 2016), visual processing in the visual periphery (Deza & Eckstein, 2016; Lago, Sechopoulos, Bochud, & Eckstein, 2020; Michel & Geisler, 2011; Rosenholtz, 2016; Semizer & Michel, 2017; Strasburger, Rentschler, & Jüttner, 2011), search inefficiencies (Lago et al., 2021; Morvan & Maloney, 2012; Verghese, 2012), and decision suboptimalities (Mitroff & Biggs, 2014; Wolfe, Horowitz, & Kenner, 2005). To mitigate search errors, humans use target features, cues, and context that are predictive of target locations to orient covert attention, and guide the foveal region of the eye toward task-relevant locations (Bravo & Farid, 2009; Castelhano & Heaven, 2010; Eckstein, 2017; Eckstein, Beutter, & Stone, 2001; Eckstein, Drescher, & Shimozaki, 2006; Findlay, 1997; Koehler & Eckstein, 2017a; Malcolm & Henderson, 2009; Neider & Zelinsky, 2006; Torralba, Oliva, Castelhano, & Henderson, 2006; Võ, Boettcher, & Draschkow, 2019; Võ, 2021; Wolfe, Võ, Evans, & Greene, 2011).

Gaze shifts of individuals in a scene (i.e. gazers) can play an important role in guiding and facilitating visual search. In these scenarios, the gazer does not typically appear in an observer's central vision. More often the gazer appears at the observer's visual periphery. Inferring the gaze direction of others in these situations requires taking into account not only the gazer's eye region, which might not be visible in the periphery (Loomis, Kelly, Pusch, Bailenson, & Beall, 2008) but the head orientation, body postures, as well as the dynamics of the head and body movements. Studies have found that the combination of eyes, head, and body influences attentional shifts. For example, studies have found a stronger effect in both orienting overt (Azarian et al., 2017) and covert attention (Bayliss et al., 2004a; Hietanen, 1999; Hietanen, 2002) when either the head orientation and eye gaze direction are incompatible (e.g. the head rotates to the right but the eyes look toward the front/left) or when the head and body orientations are incompatible (see review Frischen, Bayliss, & Tipper, 2007). The effect of covert attention at the gazed location is also more temporally sustained attention when the head and body are present (Han & Eckstein, 2022).

To our knowledge, no study has investigated the effect of dynamic gaze on multiple eye movements visual search with various gaze cue eccentricities and real-world scenes. Here, we evaluate the contributions of a gazer's head and body cues in guiding eye movements and facilitating the search for a target person with videos of real scenes. If the gazer is looking to the left side of the image while the gaze goal (the designated gazed person) is on the opposite side, then we should expect the gaze-following eye movements might impact visual search. The results could shed light on the connection between available gaze information in the video, active eye movement planning, and behavioral performance in visual search tasks.

**Figure 1.**
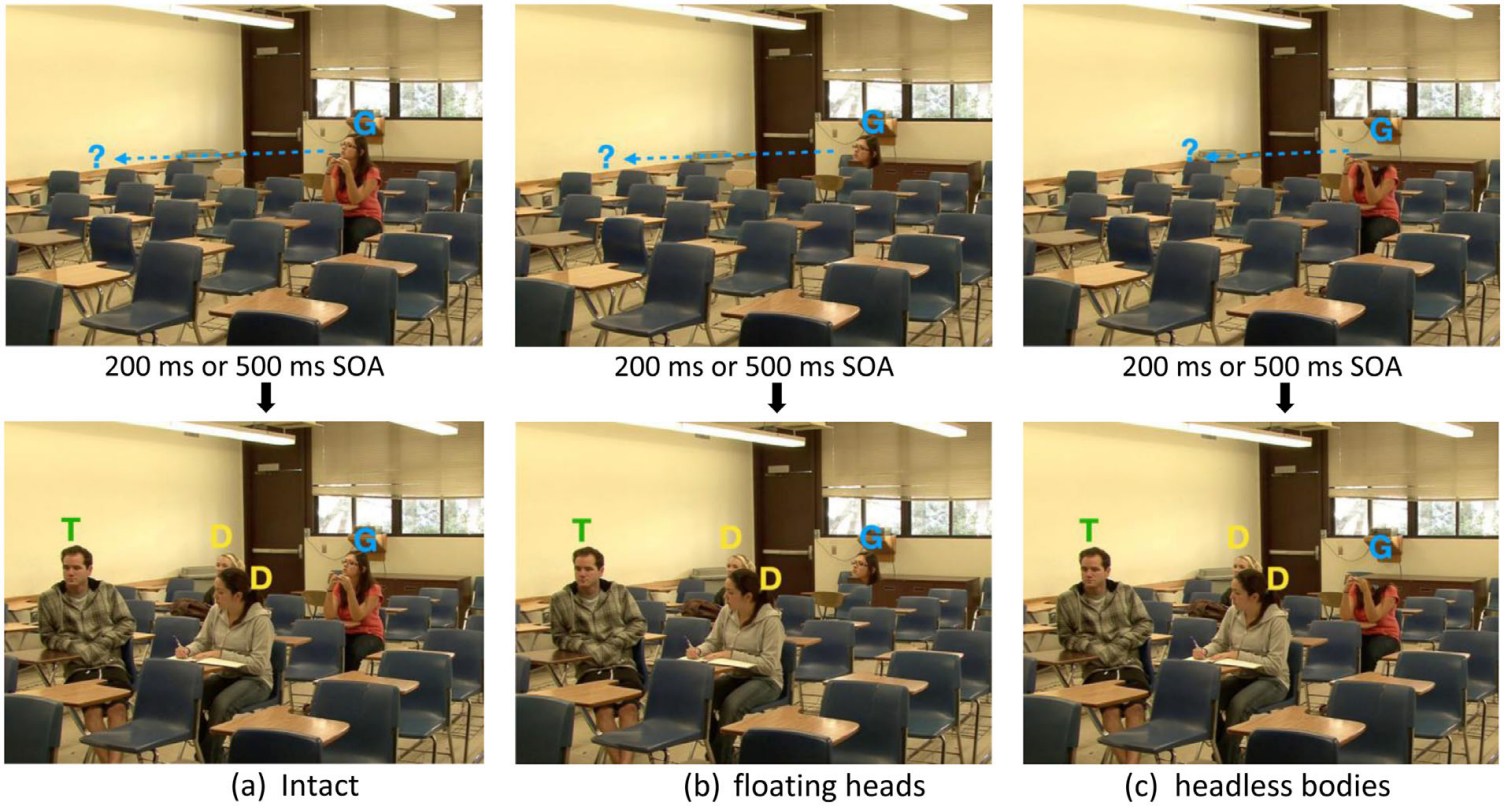
Example frames from videos with a valid gaze cue. In the videos, the gazers (G) looked at the same designated person (gaze goal). After 200 ms or 500 ms, the target person (T) and some other distractor persons (D) appeared in the video. The letter notations were not included in the actual video stimuli during the experiment and are only presented here for illustration purposes. (**a**) Intact condition: gazer (G) is intact (**b**) floating heads condition: gazer (G) has floating heads without a body. (**c**) Headless body condition: gazer (G) has headless bodies. Invalid gaze cue videos were similar except that the gazers looked at a location where a distractor individual rather than the target was presented.

In addition, we used digital video editing techniques to erase the head or lower body of the gazers and replace them with the immediate background and to create three different experimental conditions (gazer intact, floating heads, and headless bodies; Figure 1). This experimental manipulation allowed us to isolate the effect of gazers’ head and lower body movements separately on eye movements during visual search. At the beginning of the videos, only the gazers’ behaviors were visible to the observers to make sure the eye movements planning was only dependent on the gaze information. After the gaze behavior stopped and observers were allowed to make eye movements to search for the target, we then showed the multiple distractor people along with the target (only in the target present condition). If only one person (either a distractor or the target) appeared suddenly after gaze behaviors were completed, the sudden onset could naturally interrupt natural eye movements and attract the observer's attention regardless of whether the observer was following the gaze direction or not. Therefore, we presented multiple distractors rather than just a single individual for the search task.

To further explore how eye movement planning during gaze-following was related to available gaze information (e.g. direction of the head and lower body) in the videos, we collected a separate dataset where people made explicit judgments about the location at which gazers’ looked at (gaze goal). We considered these judgments as estimates of the upper limits of the information available to the saccade system to guide eye movements during the search. By comparing the perceptual judgments of gaze goals to the eye movements during the search, we could assess whether the eye movement errors with various head/body cues are related to the inherent perceptual information about gaze-goal available in the head/body of gazers.

## Materials and methods

### Search task

#### Subjects

Twenty undergraduate students (aged = 18–21, 12 women and 8 men) from the University of California, Santa Barbara, CA, were recruited as subjects for course credits in this experiment. All had normal to corrected-to-normal vision. All participants signed consent forms to participate in the study. Individuals filmed in the videos signed consent forms authorizing the presentation of their images in the scientific publication and presentations of the study. The study was approved by the Institutional Review Boards at the University of California, Santa Barbara, CA.

#### Stimuli and instruments

The stimuli consisted of 60 clips from in-house videos (approximately 3 seconds in length) that were originally filmed at the University of California, Santa Barbara, CA, campus. Videos included both indoor and outdoor scenes, such as classrooms, outside campus buildings, dining halls, etc. In each video clip, there were multiple students instructed during filming to look toward a designated person at the same time. We refer to the individuals in the videos shifting their gaze as “gazers.” The designated gazed person (“gaze goal”) could be the target person that observers were looking for (50% of trials), or could be a distractor person (the remaining 50% of trials). The target person was the same individual across all videos and subjects (see Figure 1). The videos were filmed on different days. Thus, the target person could appear with different clothing across the videos.

**Figure A1.**
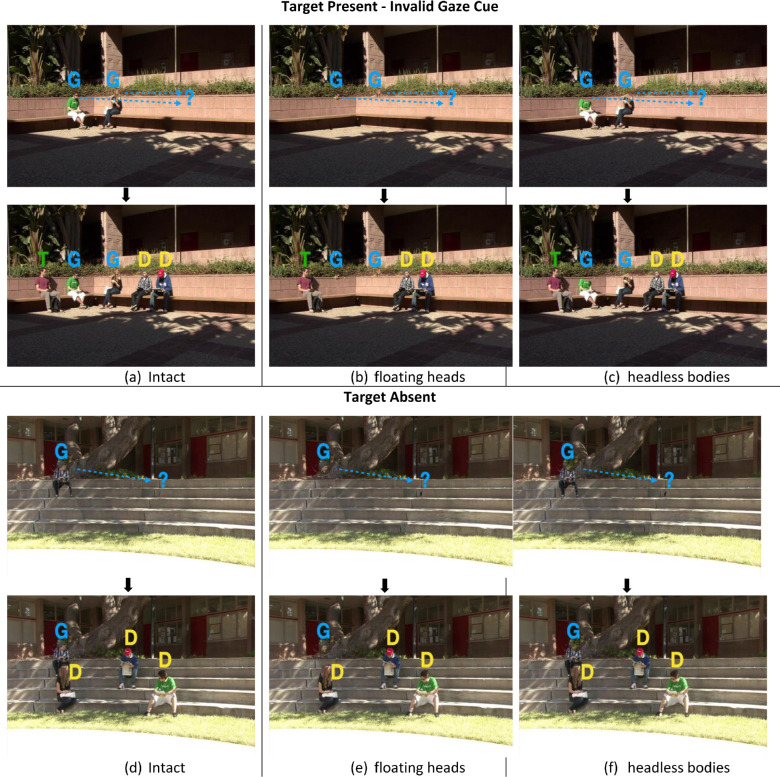
Example frames from three conditions. Gazers (G), Target (T), Distractor (D). (**a-c**) Target present with an invalid cue: Gazers (G) gaze at the location with the target (T) and two distractors (D) showing up after a 200 ms or 500 ms delay. In this case, a distractor (D) instead of the target (T) shows up at the gaze goal location, so the cue is invalid. (**d-f**) Target absent: Gazers (G) gaze at the location with three distractors (D) showing up after a 200 ms or 500 ms delay.

For each video clip, we first extracted individual frames. Then, we manually segmented each individual's head and body outlines. We randomly selected some gazers to be digitally deleted during the initial presentation of the video (gaze cueing process), along with the gaze goal person (person who was gazed at by all the gazers). In order to do this, we picked out the frames for the gaze-orienting process and replaced the red, green, and blue (RGB) values of pixels contained by the outline of the individuals to be deleted with the RGB values of those pixels of the immediate background (available from other frames without the individuals). This method allowed us to delete some gazers and the target/distractor individually from the initial portion of the video frames prior to the end of the gazers’ head movement. By changing the selected gazers, we were able to create multiple versions of a video. For each video, the gaze goal person and the distractors were digitally deleted from the video frames, and appeared either 200 ms or 500 ms (stimulus onset asynchrony [SOA]) after the completion of the gazer's head movements. Finally, processed frames were compiled to create videos that have only one to three individuals orienting their gaze, heads, and bodies toward a point in the scene, followed by the appearance of two to four individuals (target and/or distractor individuals) after a 200 or 500 ms delay (see Figure 1). See Figure A1 for more video frame examples for invalid cue or target absent. Out of all the videos, 38% had the same number of distractors on each side of the image (left versus right of the central fixation), 28% had the majority of distractors on the left side, and 33% had the majority of distractors on the right side. In addition, for those videos which had an imbalanced number of distractors on two sides, there was no relationship between the gazed target person's location (image side) and the majority of distractors (χ^2^ = 0.75, *p* = 0.39). Therefore, there was no bias in the distractor locations that could be used by participants as a cue to orient eye movements and guide eye movements toward the target.

In addition to the condition where gazers’ heads and bodies were present (intact condition), we also created another two conditions referred to as floating heads and headless bodies, where gazers’ bodies or heads were digitally deleted during the gaze-orienting process, respectively, and replaced by the background (see Figures 1b, c). Different conditions manipulated the head/body features present in the videos for the gazers (but not the target/distractor individuals). In summary, we created videos for three conditions: (1) intact videos, (2) floating head videos (gazers’ bodies were invisible), and (3) headless body videos (gazers’ heads were invisible). In all videos, we retained the immediate background behind the erased heads or bodies (see Figure 1). See Figure A1 for more examples.

All videos were presented at the center of the computer screen with a visual angle of 18.4 degrees × 13.8 degrees (width x height). Participants’ eyes were 75 cm away from a Barco MDRC 1119 monitor (1280 × 1024 pixels). All participants’ left eyes were tracked by a video-based eye tracker (SR Research Eyelink 1000 plus Desktop Mount) with a sampling rate of 1000 Hz. Their eye movements were calibrated and validated before the experiment. Events in which velocity was higher than 35 degrees/second and acceleration exceeded 9500 degrees/second² were recorded as saccades.

#### Procedure

**Figure 2.**
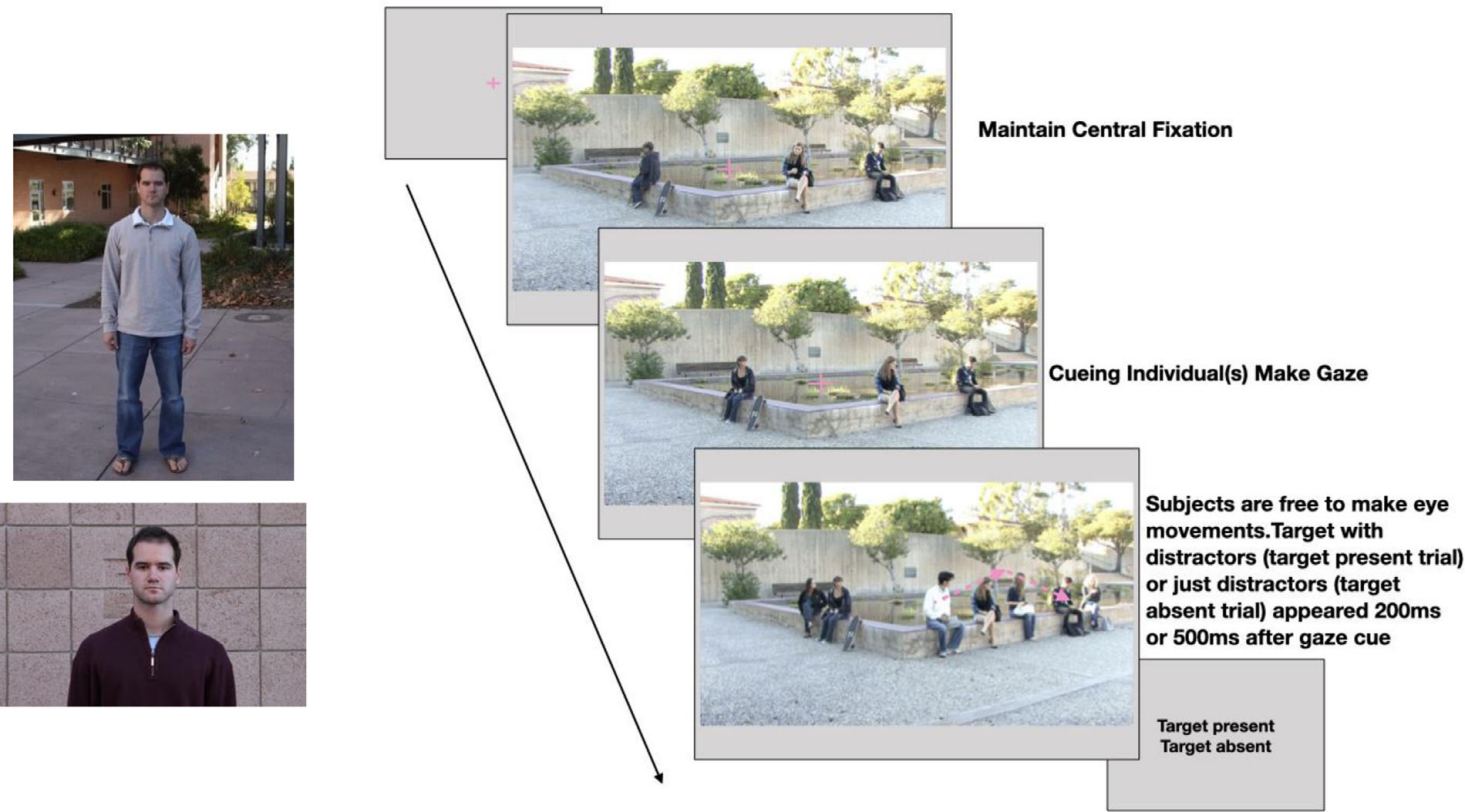
(**a**) Photograph of the target person and an accompanying close-up shot. Participants were shown the target person's photos on the response screen. (**b**) Timeline for each trial. The participants were required to fixate at the center cross and press the space bar to initialize the trial. The video started with one or multiple gazers looking at the same person. Once the gazer's head/body movement ended, the central cross disappeared, and observers were free to make eye movements. Either 200 ms or 500 ms after the disappearance of the central cross, other individuals (target with distractors or distractors only) appeared in the video for 1000 ms. Participants indicated whether the target person was present or absent in the video. Observers were free to execute eye movements and were given no instructions related to search strategies.

Subjects were asked to judge whether a target person was present or absent in the videos. The target was a specific person present in 50% of the videos. Observers were first given unlimited time to familiarize themselves with pictures of the target person in different clothing outfits (see Figure 2a). Then, they completed a practice session with 10 videos in order to make sure they were able to identify the target person. There was only one target person across all trials. These practice videos were different from the videos for the main experiment.

Participants then proceeded to complete the main experiment with three conditions (1 = intact; 2 = floating heads; and 3 = headless bodies) in a random blocked order within one sitting. Videos were presented randomly within each block (condition). Each session included a complete set of all three conditions. Each subject finished two sessions, thus resulting in 360 trials (60 trials/condition × 3 conditions/session × 2 sessions) total. Participants were required to complete the eye tracker nine-point calibration and validation before the experiment started. Before the initiation of a trial, observers would recalibrate and revalidate if there were any large eye drifts detected that caused failure in maintaining fixation (>1.5 degrees visual angle).

On each trial, the participants were instructed to fixate on the central cross while pressing the space bar to start the video. Once the video started, the central cross stayed on the screen, and participants were instructed to fixate on the cross without eye movements while gazers shifted their gaze. If an eye-position deflection greater than 1.5 degrees visual angle from the fixation cross was detected during the gaze shift, that trial was aborted. At the moment when all the gazers looked at the designated person and stopped moving, the central cross disappeared, and observers were free to make eye movements. Either 200 ms or 500 ms delay after the central cross disappeared, the target with distractors (target-present trials) or all distractors (target-absent trials) were digitally re-inserted into the videos for 1000 ms before the response screen (see Figure 2b).

Finally, participants made the response by pressing a key to indicate whether the target person was present or absent (see Figure 2b). Pictures of the target person were presented for reference when they made a response after each video.

### Explicit gaze estimates task

#### Subjects

One hundred subjects (age over 18 years) were recruited with a human interface task (HIT) posted on Amazon Mechanical Turk (Mturk). The study was approved by the Institutional Review Boards at the University of California, Santa Barbara, CA. All subjects consented to participate in the experiment.

#### Stimuli

Stimuli consisted of individual frames from the same videos presented in the eye-tracking search experiment. Specifically, for each of the 60 videos in each condition (intact, floating heads, and headless bodies), the frame in which all gazers directly looked at the same person was extracted. However, the gazed-at person was deleted from the frame and was replaced by the background pixel values to produce images with minimal visible manipulations to the observers. There were 180 different images (60 videos × 3 conditions) in total.

#### Procedures

Subjects were asked to make an explicit perceptual estimation of a gazer's goal by selecting locations on the image where they thought all the gazers were looking. Subjects were informed that the gaze goal target was removed from each image. Their task was to make the best judgment about where the gazers were looking within the scene. Sixty images from 60 videos were presented in random order for each subject. Subjects had unlimited viewing time. Motor errors in the spatial selection of the gaze goal location could be corrected before proceeding to the next image. For each image, the condition (intact, floating heads, and headless body) of the image was selected randomly on each trial. Importantly, each subject could only see an image in a single condition to prevent interference from memory from multiple viewings of an image.

### Data analysis

#### Eye movements of search task

We used a within-subject 3 × 2 × 2 ANOVA to measure the effects of condition (intact, floating heads, and headless bodies), cue validity (valid and invalid), and SOA (200 ms and 500 ms) on the first saccade latency, fixation distance toward the target person. We used bootstrap tests to evaluate if participants were more often to make fixations on the same side of the gaze goal (follow gaze cue), and the proportion of trials where they foveated on the gaze goal/gazers (within 2 degrees visual angle). All *p* values for Tukey post hoc tests were corrected using false discovery rate (FDR).

#### Comparison of eye movements and explicit gaze goal estimates

In order to measure the relationship between the spatial distribution of eye movements and explicit perceptual judgments about gaze goals, we created fixation maps from the eye movements from the search task and perceptual judgment maps from spatial selections of the explicit gaze estimates study. For each video in the eye-tracking experiment, all subjects’ fixations were collected to create a fixation map. Similarly, for each image from the Mturk experiment, all subjects’ spatial selections were collected to create a perceptual judgment map. The fixation and gaze goal estimate maps were all smoothed with a Gaussian filter (standard deviation of 20 pixels) and were then normalized to sum to one. We measured the similarity of the fixation and gaze estimate maps for a video by taking the normalized dot product of the maps. We used permutation tests (10,000 permutations) across videos for the pairing of fixation and perceptual judgment maps to obtain a distribution of dot products that one might expect by chance. To assess differences in eye movement distributions across different conditions (intact, floating heads, and headless bodies), we also ran 1-way ANOVA tests on the dot product between intact gaze goal estimate maps and the fixation maps from all three conditions.

#### Target detection performance for search task

We examined the effect of gaze cueing on search detection performance. We conducted a three-way within-subject ANOVA to test the effects of condition (intact, floating heads, and headless bodies), cue validity (valid and invalid), and SOA (200 ms and 500 ms) on the hit rate (proportion of correctly detected target-present trials). We conducted a within-subject 2-way ANOVA (condition × SOA) on false positive rates. We corrected the *p* values using FDR for the Tukey post hoc tests to reduce the probability of making a type I error. Multiple one-sample *t*-tests were implemented to test the significance of ∆d’ (valid d’ – invalid d’) with FDR correction. Then a 1-way ANOVA on sensitivity difference ∆d’ was conducted to test differences among the three conditions.

#### Relationship between eye movements and performance for search task

Finally, we computed a correlation between the cueing effect for behavioral performance (mean difference, valid-invalid, in sensitivity d’) and the cueing effect for eye movements (the mean difference, invalid-valid, in the distance of the closest fixation to the target).

### Results

#### First saccade latency is affected by SOA delay

**Figure 3.**
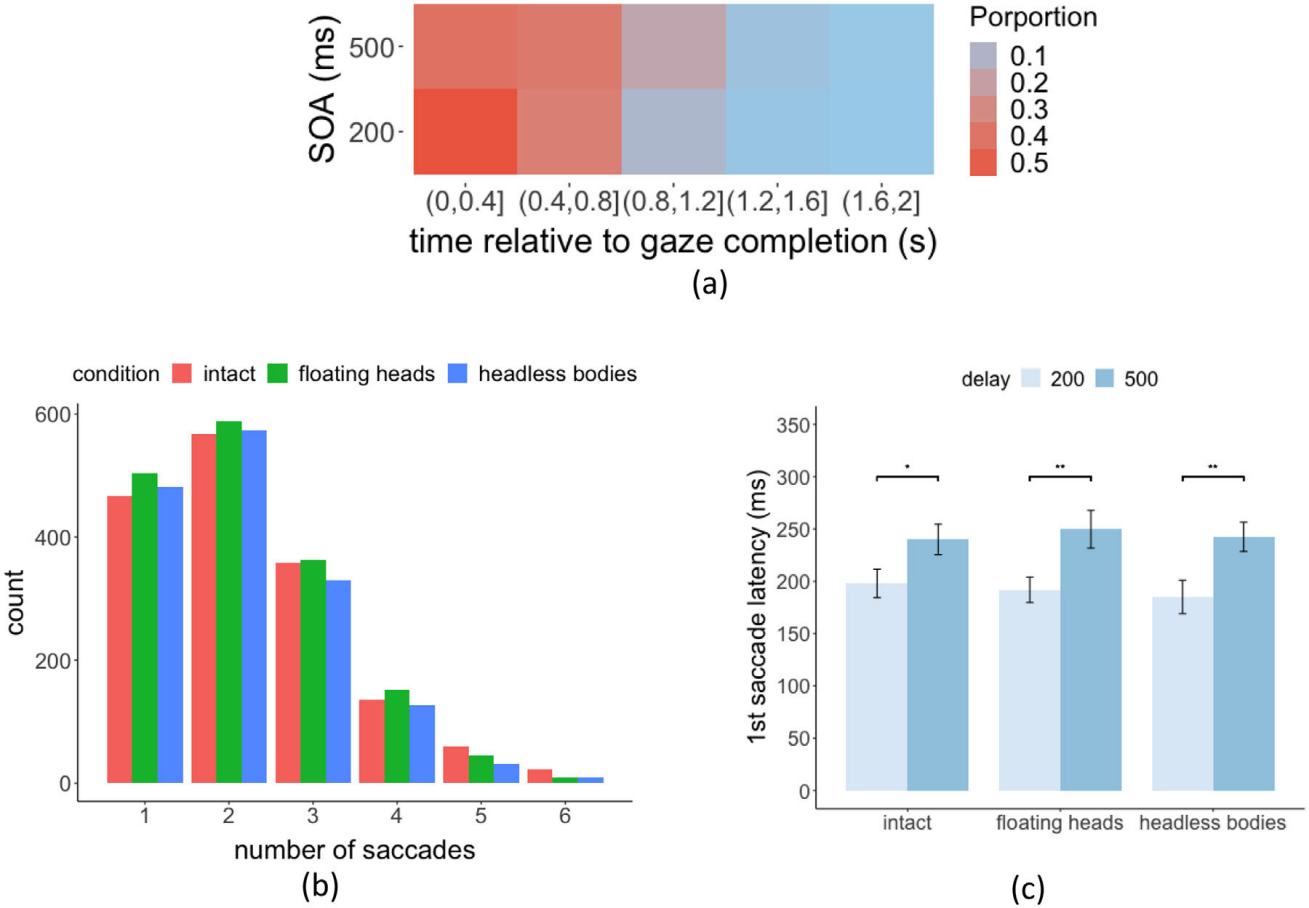
(**a**) The frequency of the first saccade starting time relative to the time the gazer's head movement stopped (**b**). Histogram of the number of saccades made during 1000 ms target/distractor presentation (**c**). First saccade latency for 200 ms and 500 ms SOA delay.

In most trials, observers made two to three saccades within 1000 ms when they searched for the target in the videos (Figures 3a,b). A three-way (condition × SOA × cue validity) within-subject ANOVA showed that there was a main effect of SOA (200 vs. 500 ms) on the first saccades’ latency F(1, 19) = 27.56, *p* = 4.56e-05 (Figure 3c). No effect of cue validity (F(2, 38) = 2.0, *p* = 0.15) or condition (F(2, 38) = 0.26, *p* = 0.78) was found on the first saccade latency. The FDR post hoc corrections showed significantly longer saccade latency in trials with longer SOA delay than that with shorter SOA delay in all three conditions. In the intact condition, 500 ms SOA, m = 240.1 ms, SE = 10.6 ms; 200 ms SOA, m = 198.0 ms, SE = 10.11 ms, *p* = 0.047; in the floating heads condition, 500 ms SOA, mean = 249.7 ms, SE = 12.1 ms; 200 ms SOA, m = 191.8 ms, SE = 9.4 ms, *p* = 0.002; and, in the headless bodies condition, 500 ms SOA, m = 242.5 ms, SE = 10.6 ms versus 200 ms SOA, m = 185.0 ms, SE = 10.9 ms; *p* = 0.0021. Thus, subjects took longer to execute the first saccades after processing the gaze cueing while fixating at the central cross when the target/distractor people appeared with a longer SOA delay.

#### Fixations are guided by gaze cues

**Figure 4.**
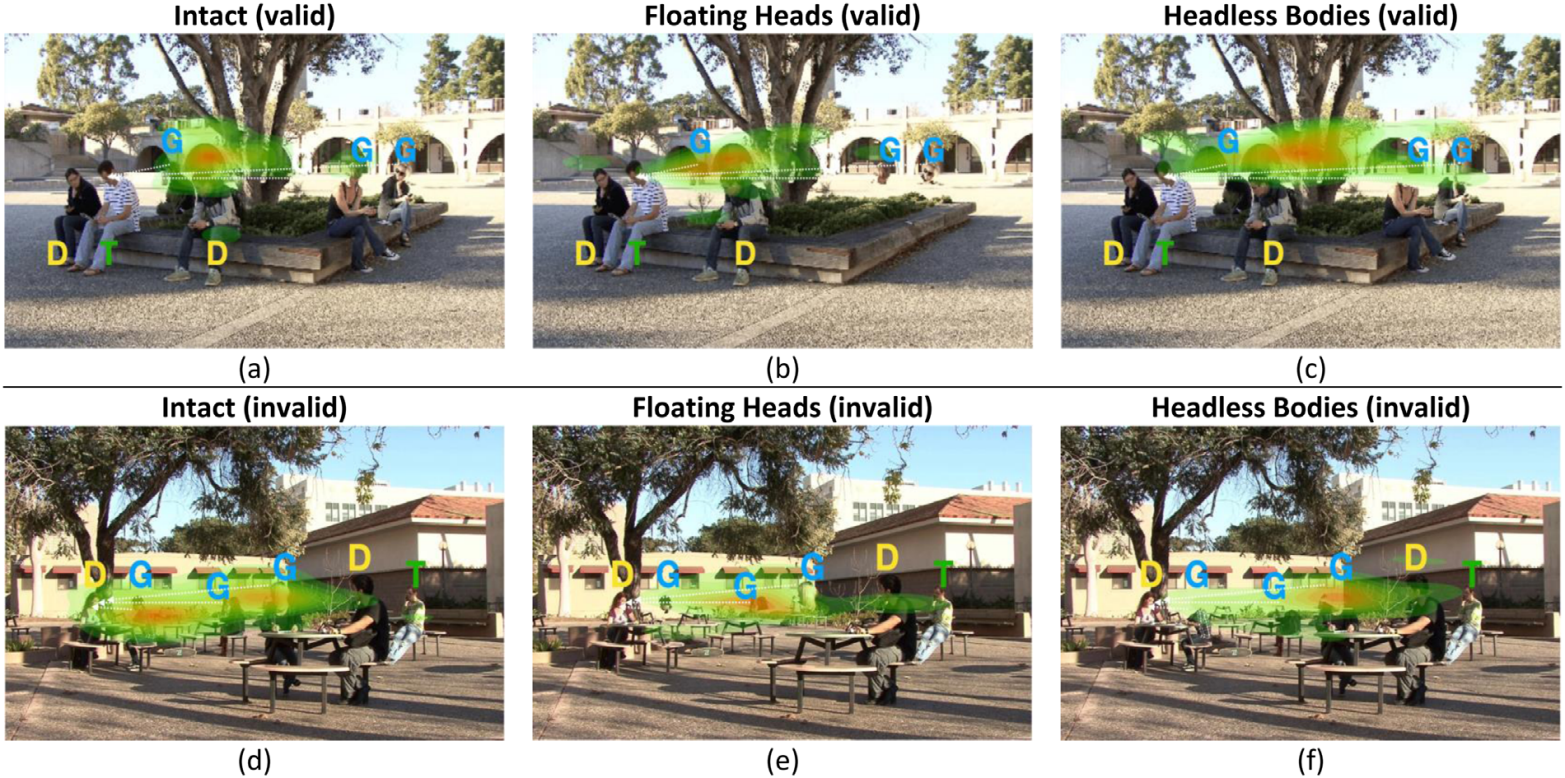
Examples of first fixations heatmaps, where G stands for the gazer, D stands for distractors, T stands for target person, and *dashed lines* show the gaze direction. Note that we have different versions of gazers for each video that were randomly selected during the experiment. (**a**) Intact condition, valid cues, (**b**). Floating heads condition, valid cues (**c**). headless bodies condition, valid cues, (**d**). Intact condition, invalid cues, (**e**). Floating heads condition, invalid cues, (**f**). Headless bodies condition, invalid cues.

**Figure 5.**
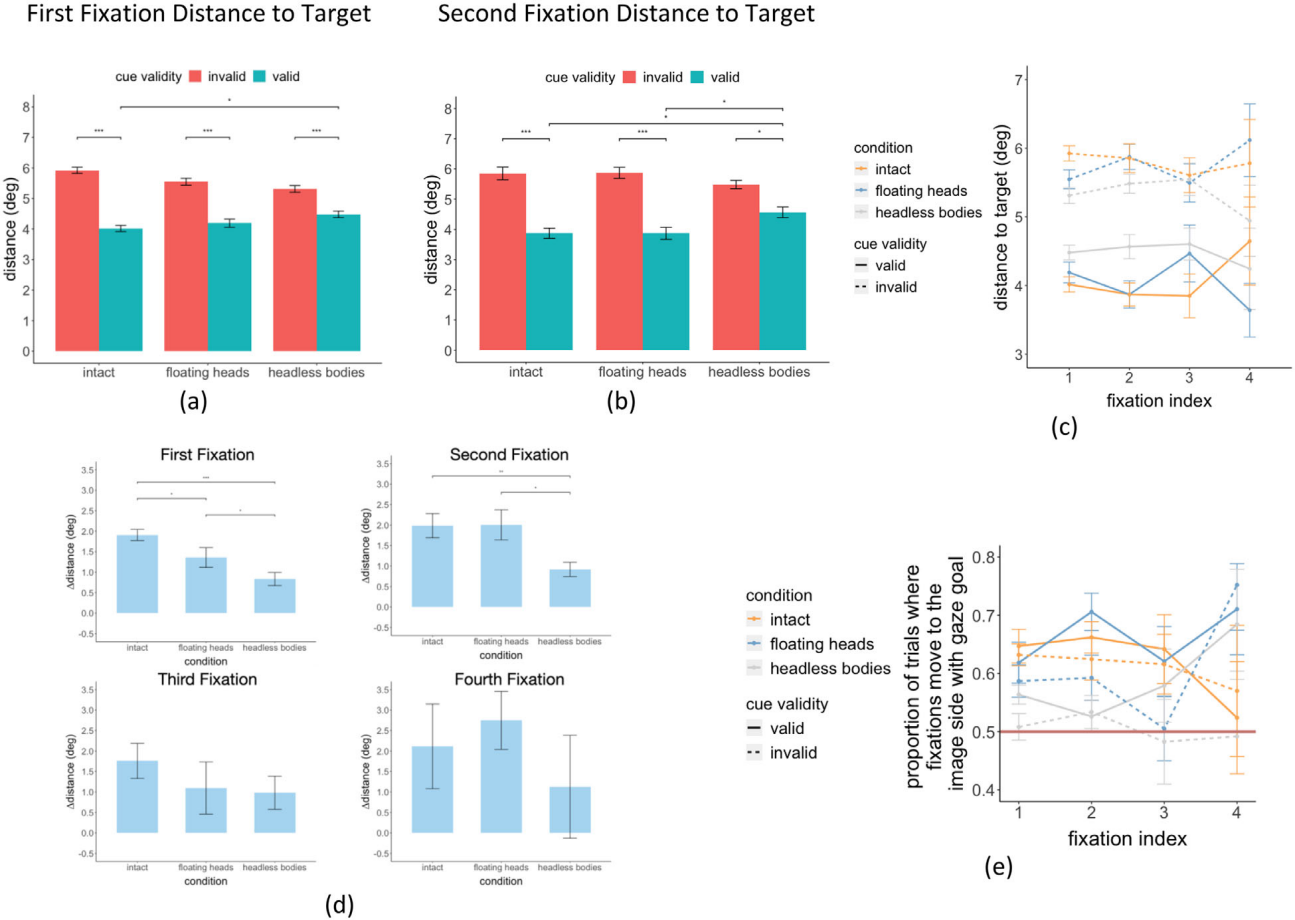
(**a,** **b**) Distance (degree) between the first and second fixations and the target person in valid and invalid trials. (**c**) The fixations (first to the fourth) distance to the target in order (fixation index). (**d**) The ∆distance (invalid-valid) between fixations (first to the fourth) and the target person. All error bars represent standard error across subjects. (**e**) The proportion of trials for fixation location on the side of the image with the gaze-goal.

We first analyzed the effect of the gaze cue in orienting the first fixation. Figure 4 shows examples of heatmaps of first fixation positions for the three conditions for valid and invalid gaze cue trials. A three-way (condition × SOA × cue validity) within-subject ANOVA showed a significant main effect of cue validity on the distance of the first fixations to the target person, F(1, 19) = 85.39, *p* < 0.001, and a significant interaction between condition and cue validity, F(2, 38) = 15.59, *p* < 0.001. The FDR corrected post hoc Tukey tests showed significantly shorter distances between the first fixation and the target person when the cue was valid versus when the cue was invalid in all three conditions (Figure 5a, Table 1 for details). With valid gaze cues, the distance to the target was significantly higher in the headless bodies condition compared to the intact condition, *p* = 0.014, but no other difference was found (intact versus floating heads, *p* = 0.80, floating heads versus headless bodies, *p* = 0.29). The length of the SOA did not affect the location of the first fixations (F(1, 19) = 0.68, *p* = 0.42).

**Table 1.** All fixation distance to the target summary (with standard errors across subjects in parentheses and *p* values below the standard errors).

	First fixation		Second fixation		Third fixation	
	Invalid	Valid	Invalid	Valid	Invalid	Valid
Intact	5.9 degrees	4.0 degrees	5.85 degrees	3.87 degrees	5.61 degrees	3.84 degrees
	(0.11 degrees)	(0.11 degrees)	(0.21 degrees)	(0.17 degrees)	(0.25 degrees)	(0.32 degrees)
	***p* = 1.95e-11**		***p* = 2.09e-06**		***p* = 1.0e-04**	
Floating heads	5.55 degrees	4.19 degrees	5.87 degrees	3.87 degrees	5.49 degrees	4.47 degrees
	(0.14 degrees)	(0.15 degrees)	(0.18 degrees)	(0.20 degrees)	(0.28 degrees)	(0.42 degrees)
	***p* = 2.11e-05**		***p* = 2.94e-05**		***p* = 0.0075**	
Headless bodies	5.3 degrees	4.5 degrees	5.48 degrees	4.57 degrees	5.55 degrees	4.60 degrees
	(0.11 degrees)	(0.11 degrees)	(0.14 degrees)	(0.18 degrees)	(0.24 degrees)	(0.23 degrees)
	***p* = 4.77e-05**		***p* = 4.41e-05**		***p* = 0.045**	

All *p* values for Tukey post hoc tests were corrected using false discovery rate (FDR). BOLD *p* values were significant.

Similarly, we found a significant main effect of cue validity on the second fixation's distance to the target person, F(1, 19) = 59.96, *p* = 2.71e-07), and a significant interaction between the cue validity and body part condition, F(2, 38) = 6.46, *p* = 0.004 (Figure 5b). Post hoc tests showed a higher distance to the target when the cue was invalid in all three conditions (see Table 1 for details). In addition, given a valid cue, distance to the target was highest in the headless bodies condition compared to both the intact condition, *p* = 0.028, and the floating heads condition, *p* = 0.028, but no difference between the intact and floating heads, *p* = 1.00. The cueing effect on eye movement guidance toward the target persisted for the third fixations (see Table 1 for details). For the fourth fixations, there were fewer trials, and we did not observe any significant effect (see Figure 5c for the first to the fourth fixation across all conditions).

To further quantify the influence of the gaze cue in guiding eye movements among the three conditions, we calculated the difference between the fixations’ distance to the target between cue valid and invalid (∆distance = distance invalid – distance valid; Figure 5d). One-way ANOVA on ∆distance for the first fixation showed a main effect of condition for the first fixation, F(2, 38) = 15.59, *p* = 1.14e-05. FDR-corrected post hoc tests showed a significantly higher ∆distance in the intact condition compared to that of the floating heads condition, *p* = 0.024, as well as compared to that of the headless bodies condition, *p* = 1.42e-06 (see detailed results in Table 2). In addition, ∆distance in the floating heads condition was significantly higher than that of the headless bodies condition, *p* = 0.024. A similar result was found for the second fixation, F(2, 38) = 6.46, *p* = 0.0038, but no main effect of condition was found for the third fixation, F(2, 55) = 0.72, *p* = 0.49, and fourth fixation, F(2, 37) = 0.69, *p* = 0.51 (see Table 2, Figure 5d).

**Table 2.** Mean ∆distance (invalid-valid) to the target for all the conditions and fixations, with standard errors across participants, and *p* values for comparisons against 0 (corrected by FDR).

	First fixation	Second fixation	Third fixation	Fourth fixation
Intact	1.91 degrees	1.98 degrees	1.76 degrees	2.11 degrees
	(0.14 degrees)	(0.30 degrees)	(0.43 degrees)	(1.03 degrees)
	***p* = 2.92e-11**	***p* = 3.14e-06**	***p* = 0.0009**	***p* = 0.048**
Floating heads	1.36 degrees	2.00 degrees	1.10 degrees	2.75 degrees
	(0.24 degrees)	(0.37 degrees)	(0.64 degrees)	(0.71 degrees)
	***p* = 1.58e-05**	***p* = 2.20e-05**	*p* = 0.0507(*)	***p* = 0.002**
Headless bodies	0.83 degrees	0.91 degrees	0.98 degrees	1.13 degrees
	(0.16 degrees)	(0.17 degrees)	(0.40 degrees)	(1.26 degrees)
	***p* = 2.38e-05**	***p* = 2.21e-05**	***p* = 0.019**	*p* = 0.19

BOLD p values were significant.

To further understand whether the first eye movement was indeed directed toward the gaze goal, we calculated the proportion of trials where the fixation was located on the same side (left/right) as the gaze goal (note that the starting point was always the fixation cross at the center of the image; Figure 5e). We found that the proportion of first fixations directed to the side of the image with the gaze goal was significantly greater than 0.5 (bootstrap test with 10,000 samples, detailed results in Table 3) indicating that the participants were indeed following the gaze direction most times. The one exception was the invalid cue trials in the headless bodies condition which was not significantly different than 0.5 (*p* = 0.36).

**Table 3.** The proportion of trials where 1st fixation was located at the same side (left/right) as the gaze goal, and bootstrap *p* values for comparisons against 50% (corrected by FDR).

	Cue validity	Proportion	*p* value
Intact	Invalid	63%	***p* < 1e-05**
	Valid	65%	***p* < 1e-05**
Floating heads	Invalid	59%	***p* = 6.0e-04**
	Valid	62%	***p* = 8.4e-04**
Headless bodies	Invalid	51%	*p* = 0.36
	Valid	56%	***p* < 1e-05**

BOLD *p* values were significant.

**Figure 6.**
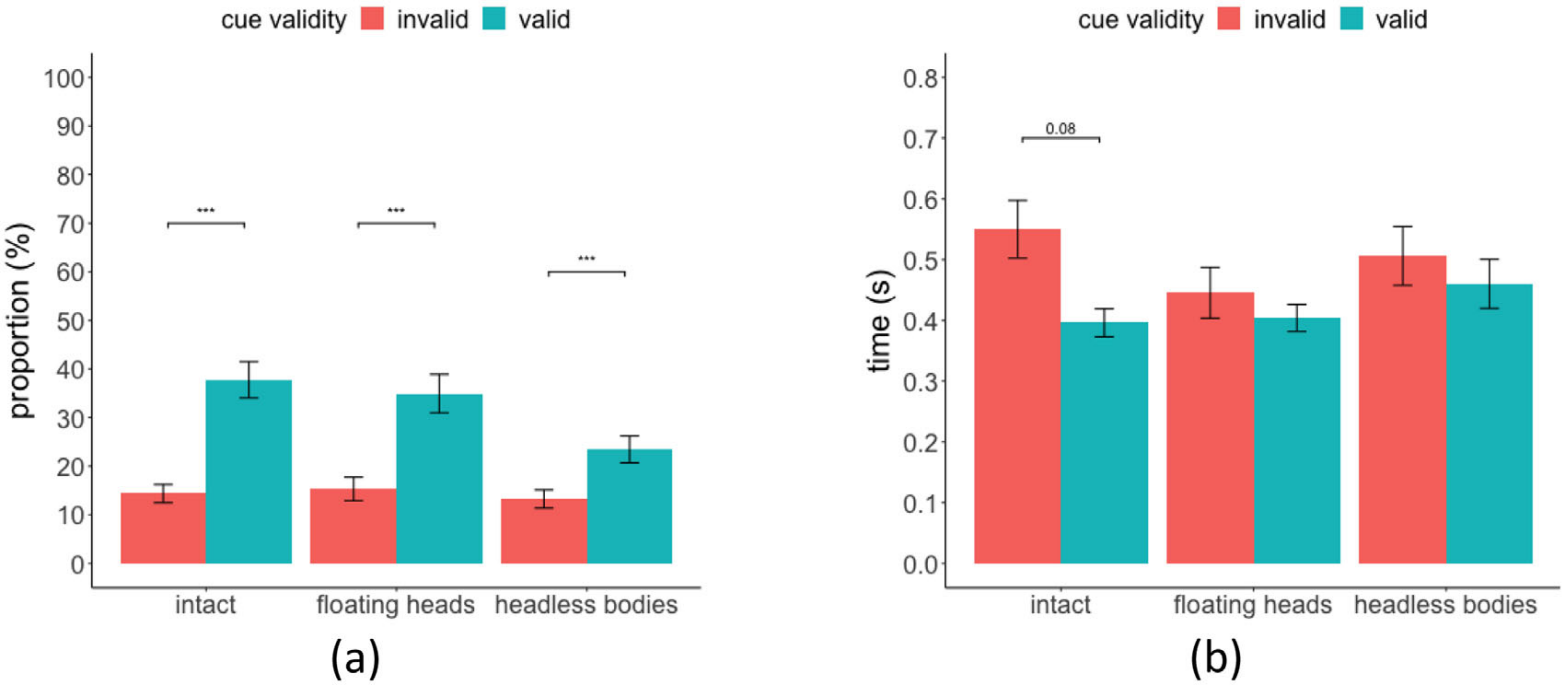
(**a**) The proportion of trials with saccades foveating (within 2 degrees) at the target person's head. (**b**) The average time it took for participants to foveate (within 2 degrees) the target person's head.

**Figure A2.**
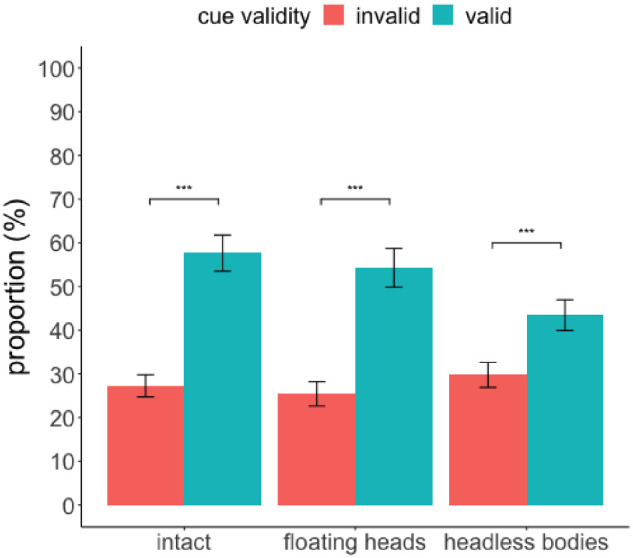
The proportion of trials foveating at the target (within 3 degrees of the visual angle).

A two-way (gazer's body parts/whole condition times cue validity) within-subject ANOVA test on the percentage of trials in which subjects foveated the target person's head (within 2 degrees visual angle) showed a significant main effect of body parts/whole condition F(2, 38) = 11.09, *p* = 1.61e-04, cue validity F(1, 19) = 38.55, *p* = 5.78e-06. There was also a significant interaction between the gazer's body parts/whole condition and cue validity F(2, 38) = 6.24, *p* = 0.005. FDR corrected post hoc test showed a statistically significantly higher proportion of trials with fixations foveating on the target person in the valid trials versus invalid trials in intact (valid = 37.77%, SE = 3.73%, invalid = 14.35%, SE = 1.88%, *p* = 7.83e-06), floating heads condition (valid = 34.94%, SE = 3.97%, invalid = 15.31%, SE = 2.42%, *p* = 3.98e-04), as well as in the headless bodies condition (valid = 23.45%, SE = 2.75%, invalid = 13.23%, SE = 1.86%, *p* = 2.67e-05; Figure 6a). Similar results can be found if we adjust our criterion of foveation from within 2 degrees to within 3 degrees of the target (see Figure A2). For the subset of trials in which participants foveated at the target person, we also found a significant main effect of cue validity on the time it took to foveate the target, F(1, 110) = 4.2, *p* = 0.043 (Figure 6b). The post hoc test showed a trend for longer times for participants to foveate the target with invalid gaze cues in the intact condition, but did not reach significance (invalid 0.55 seconds versus valid 0.40 seconds, *p* = 0.08; Figure 6b). No difference was found in the other conditions (floating heads, invalid = 0.45 seconds, valid = 0.40 seconds, *p* = 0.14; headless bodies, invalid = 0.51 seconds, valid = 0.46 seconds, *p* = 0.65).

#### Relationship between eye movement guidance and explicit perceptual estimates of gaze goals

We first quantified the perceptual estimation errors (data from the explicit gaze estimates task) for the intact, floating heads, and headless bodies conditions using the Mturk-collected perceptual judgment maps of images with the gaze goals digitally deleted. This allowed us to assess the inherent information about gaze goal that observers can perceptually extract from the heads, lower bodies and their joint presence (intact). The subjects making these perceptual estimates were different than the observers participating in the search task.

**Figure 7.**
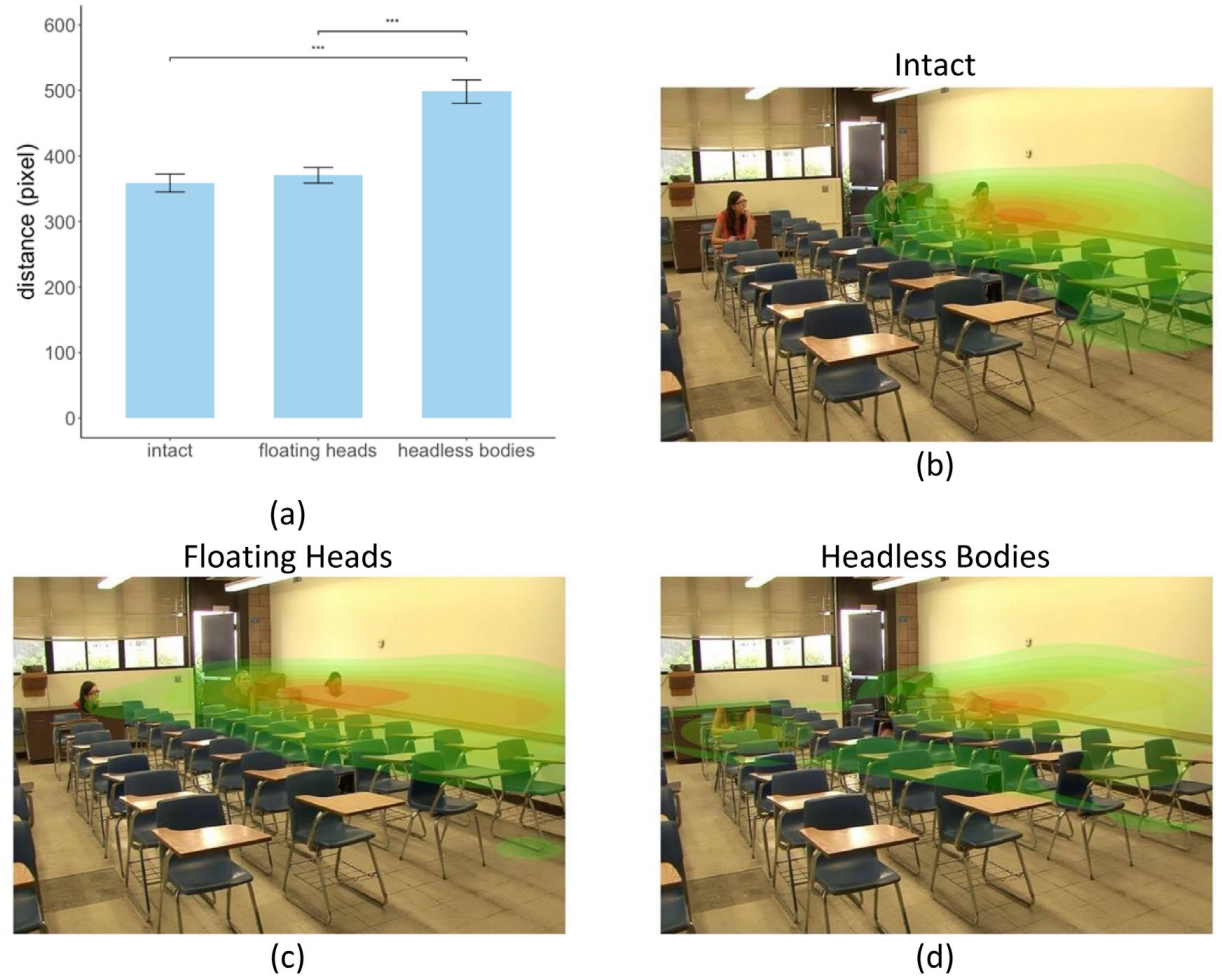
(**a**) Perceptual estimation error of the gaze goal location. It is defined as the pixel distance between the centroid of all subjects’ clicks to the ground truth gaze goal location (the head centroid position of the person who was gazed at), averaged across images. (**b**) Examples of heatmaps of perceptual estimation of gaze location from clicks, regions with a lower density of clicks were *green*, and regions with higher density were *red* (**b**) intact gazers, (**c**) floating heads gazers, and (**d**) headless bodies gazers.

As an error metric for the perceptual judgments, we calculated the distance of the peak of the density map of the perceptual estimates to the known gaze goal (location the gazer was looking at). A 1-way ANOVA found a significant main effect of body parts/whole condition on perceptual estimation error F(2, 150) = 27.76, *p* = 5.54e-11. FDR corrected post hoc tests showed statistically significant higher error in the headless bodies condition (error = 498.19 pixels and SE = 17.79 pixels) than that in the intact (error = 358.83 pixels and SE = 13.67 pixels), *p* = 9.31e-10, and the floating heads (error = 370.65 pixels and SE = 11.88 pixels) condition, *p* = 1.21e-08 (Figure 7). There was no difference between the intact condition and the floating heads condition, *p* = 0.57. The results indicate that the presence of heads improves gaze estimation accuracy.

In order to investigate the relationship between perceptual judgments and eye movement fixation locations, we computed a quantitative measure of similarity (normalized dot products) of fixation maps (mean fixations per image = 72, SD = 21) and perceptual judgment maps (mean estimates per image = 32, SD = 4). The matched image pairs normalized dot product for intact fixation-intact perceptual judgments, floating heads-floating heads, and headless bodies-headless bodies were 0.37, 0.38, and 0.38 correspondingly. The unmatched were 0.22 for the intact (averaged across intact-floating heads and intact-headless bodies normalized dot products), 0.24 for the floating heads (averaged across floating heads-intact and floating heads-headless bodies), and 0.2 for the headless bodies (averaged across floating headless bodies-intact and headless bodies-floating heads).

**Figure A3.**
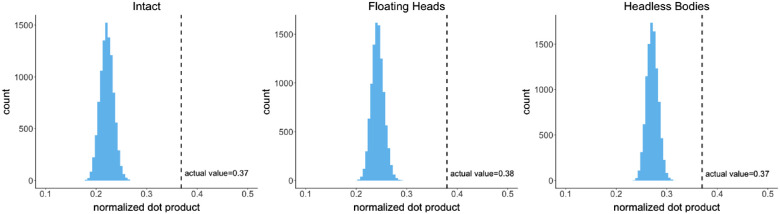
Normalized dot product permutation tests in three conditions.

The normalized dot product between fixation maps and perceptual judgment maps for the matched image pairs within each condition were significantly higher relative to the unmatched image pairs (see Figure A3 all *p* < 1e-05 based on 10,000 randomly permutated pairs). This implies, that the body parts/whole manipulations similarly influenced eye movement patterns during the search and the explicit perceptual estimations of gazer goals (under no time constraints).

**Figure 8.**
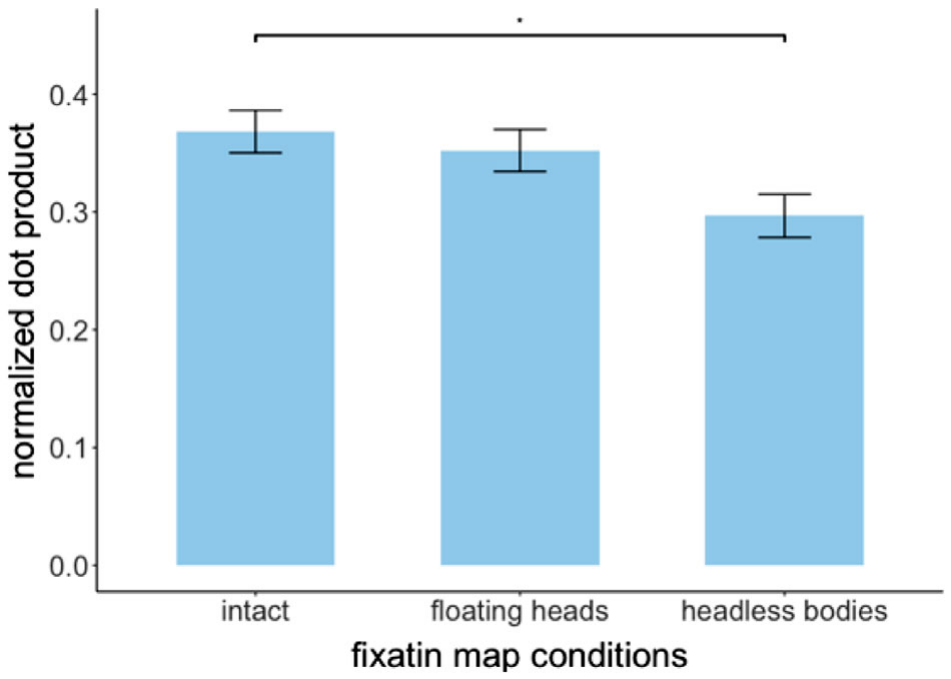
The normalized dot product between fixation maps from three body parts/whole and intact perceptual judgment map (as the benchmark).

In order to quantify differences among eye movements in the three body parts/whole conditions, fixation maps from each condition were all compared to the benchmark map of perceptual judgments of gaze goals from the intact condition. We consider the perceptual judgments of gaze goals from the intact gazers with no subject time constraints and eye movement restrictions as an upper bound of the perceptually available information about gaze goals. A 1-way ANOVA showed a main effect of body parts/whole condition on the normalized dot product between each of the three fixation maps and the perceptual judgment map from the intact condition, F(2, 177) = 4.31, *p* = 0.015. FDR-corrected post hoc tests showed a significantly higher normalized dot product between the intact fixation map and intact perceptual judgment map (normalized dot product = 0.37, SE = 0.018) compared to the headless bodies fixation map and the intact perceptual judgment map (normalized dot product = 0.30, SE = 0.018), *p* = 0.016. In addition, there was a marginally higher dot product of the floating heads fixation map and intact perceptual map than the dot product of the headless bodies fixation map and intact perceptual map (0.35 vs. 0.3), *p* = 0.08. We found no difference between the normalized dot products (computed with respect to the perceptual judgment intact condition) for the intact fixation map and the floating heads fixation (intact = 0.37, SE = 0.018 versus floating heads = 0.35, SE = 0.018, *p* = 0.81; Figure 8). These results indicated that the presence of heads led to a stronger effect in guiding eye movements toward the available perceptual information about gaze goals.

#### Eye movements strategies across multiple fixations

**Figure 9.**
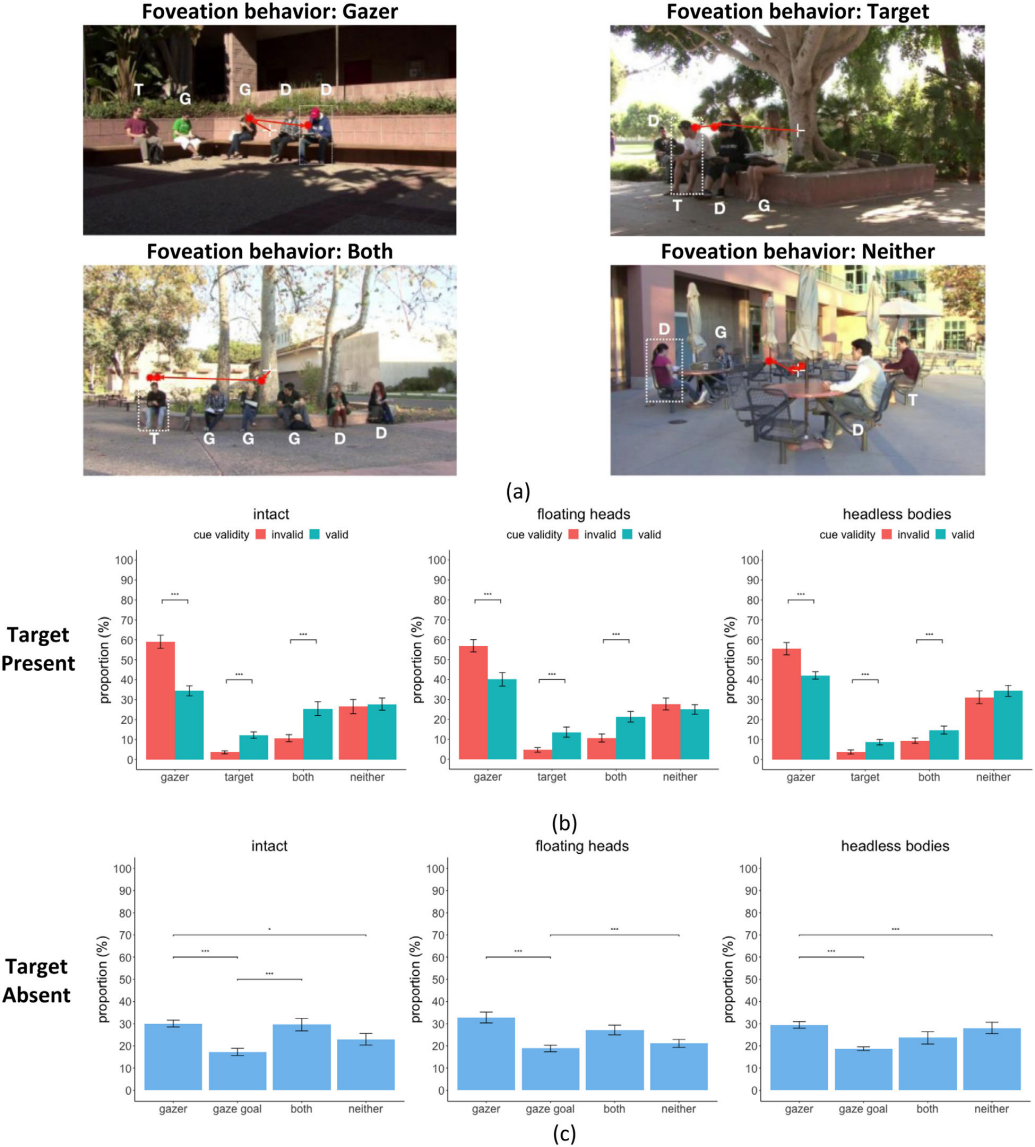
(**a**) Example fixations for four foveation behaviors. G stands for the gazer, D stands for distractors, T stands for the target person. The *red dots* represent the fixation locations and are connected in order. The person with the *white dashed box* was the gaze goal of all the gazers. Participants always started from the central fixation, and then made fixations to find the target during a 1000 ms presentation time. All the annotations are just for illustration purposes and were not present during the experiment. (**b**) The proportion of target-present trials that contained fixations foveating on: (1) only any gazers, (2) only the target, (3) both any gazers and the target, and (4) neither the gazers nor the target. (**c**) The proportion of target-absent trials that contained fixations foveating on: (1) only any gazers, (2) only the gaze goal (distractor), (3) both any gazers and the gaze goal (distractor), and (4) neither the gazers nor the gaze goal (distractor).

Our previous fixation analysis focused on the distance of the fixations to the target but did not address the possibility that observers might first fixate on a gazer and then proceed to fixate on the target or a distractor person. We further investigated trials based on fixation locations relative to gazers in the video. For trials when the target person was present, we calculated each fixation's distance to all the gazers and the target person in the video. For each fixation, we only took the distance to the closest gazer to see if the fixation was directed (within 2 degrees of the visual angle) on any of the gazers. Then, we classified the trials into four foveation behaviors: (1) gazer (trials for which the fixations were within 2 degrees of any of the gazers but not the target), (2) target (trials for which the fixations were within 2 degrees of the target but not a gazer), (3) both (trials for which the fixations were within 2 degrees of both the target and a gazer), and (4) neither (trials for which the fixations were not within 2 degrees of the target or any gazer; Figure 9a).

Then, we further calculated the proportion of each type of foveation behavior based on cue validity to evaluate the cue effects on guiding all fixations (Figure 9b). For the intact condition, we found that the proportion of trials foveating only on the target person or foveating both on the target and gazers was significantly higher for valid cue trials compared to those with invalid cues (foveating only on target, valid = 12.24%, SE = 1.62%, versus invalid = 3.62%, SE = 0.78%, bootstrap resampling, *p* < 1e-5; foveating on target and gazer, valid = 25.54%, SE = 3.46%, versus invalid = 10.73%, SE = 1.78%, *p* < 1e-5). However, there were significantly more trials containing fixations only foveating on the gazers when the cue was invalid compared to that of valid cues (invalid = 59.07%, SE = 3.30%, versus valid = 34.46%, SE = 2.52%, *p* < 1e-5), all *p* values were corrected by the FDR.

No difference across valid and invalid trials was found in the proportion of trials containing no foveations on the gazer nor target (valid = 27.76%, SE = 3.08%, versus invalid = 26.58%, SE = 3.53%, *p* = 0.31), all *p* values corrected by the FDR. Similar results were found in the floating heads and headless bodies conditions (see Table 4 for detailed results).

**Table 4.** Comparison of the proportion of trials across four foveation behaviors (with standard errors in parentheses and *p* values bold from bootstrap resampling tests) for target-present trials of valid and invalid cue trials.

	Foveation behaviors							
	Gazer		Target		Target and gazer		Neither	
	Invalid	Valid	Invalid	Valid	Invalid	Valid	Invalid	Valid
Intact	59.07%	34.46%	3.62%	12.24%	10.73%	25.54%	26.58%	27.76%
	(3.30%)	(2.52%)	(0.78%)	(1.62%)	(1.78%)	(3.46%)	(3.53%)	(3.08%)
	***p* < 1e-05**		***p* < 1e-05**		***p* < 1e-05**		*p* = 0.31	
Floating heads	56.93%	40.10%	4.67%	13.62%	10.63%	21.32%	27.77%	24.96%
	(3.12%)	(3.39%)	(1.17%)	(2.58%)	(2.02%)	(2.66%)	(2.98%)	(2.42%)
	***p* = 3e-04**		***p* = 3e-04**		***p* < 1e-05**		*p* = 0.16	
Headless bodies	55.56%	42.18%	3.77%	8.67%	9.46%	14.78%	31.21%	34.37%
	(3.09%)	(1.81%)	(1.02%)	(1.37%)	(1.31%)	(1.98%)	(3.20%)	(2.74%)
	***p* < 1e-05**		***p* < 1e-05**		***p* = 1e-04**		*p* = 0.11	

All bootstrap *p* values were corrected by false discovery rate (FDR). BOLD *p* values were significant.

Similarly, when the target person was absent, we classified the trials into the four foveation behaviors, except that we categorized the trials foveating on the gaze goal (a distractor person) rather than the target (Figure 9c). In the intact condition, a bootstrap test (10,000 samples) showed that the proportion of trials foveating only on the gaze goal person (distractor) was significantly lower compared to trials foveating only on the gazers or foveating on both the gaze goal and gazers. A similar effect was found in the floating heads and the headless bodies conditions. There was a significantly higher proportion of trials foveating neither the gazers nor the gaze goal compared to the trials foveating only the gaze goal, indicating a smaller effect in guiding eye movements with only lower body motion (see Table 5 for detailed results).

**Table 5.** Comparison of proportion of trials across four foveation behaviors (with standard errors in parentheses and *p* values in bold from bootstrap resampling tests) for target-absent trials of valid and invalid cue trials.

	Gazer	Gaze goal (distractor)	Both	Neither
Intact	30.09%	17.29%	29.59%	23.02%
	(1.54%)	(1.62%)	(2.80%)	(2.62%)
		Versus gazer**: *p* < 1e-05**		Versus gazer: *p* = 0.02
		Versus both**: *p* = 3e-04**		
Floating heads	32.82%	18.87%	27.19%	21.12%
	(2.47%)	(1.46%)	(2.20%)	(1.77%)
		Versus gazer**: *p* < 1e-05**		Versus gazer**: *p* < 1e-05**
		versus both**: *p* = 8e-04**		
Headless bodies	29.49%	18.76%	23.64%	28.10%
	(1.49%)	(0.83%)	(2.77%)	(2.55%)
		Versus gazer**: *p* = *p* < 1e-05**		
		versus neither**: *p* < 1e-05**		

All bootstrap *p* values were corrected by false discovery rate (FDR). BOLD *p* values were significant.

#### Fixation sequences

Our previous analysis focused on individual fixations. We also analyzed sequences of fixations during the visual search. We defined three types of fixation sequences: (1) look at the gaze goal: the participants only made fixations that were located within 2 degrees region of the gaze goal location; (2) look at the gaze goal, then look back at any of the gazers: participants first fixated within 2 degrees region of the gaze goal location, then fixated within 2 degrees region of any of the gazers; (3) look at the gaze goal, look back at any of the gazers, then search further: participants first fixated within 2 degrees region of the gaze goal location, then within 2 degrees region of any of the gazers, then fixated at locations outside 2 degrees region of any of the gazers to search further for the target person (Table 6).

**Table 6.** The proportion of trials that contained different types of fixation order.

Type of fixation sequences	Invalid	Valid
Look at the gaze goal	66%	66%
Look at the gaze goal, look back at gazer	22%	27.5%
Look at the gaze goal, look back at gazer, search further	11.7%	6.4%

#### Improved behavioral target detection performance with valid gaze cues

**Figure 10.**
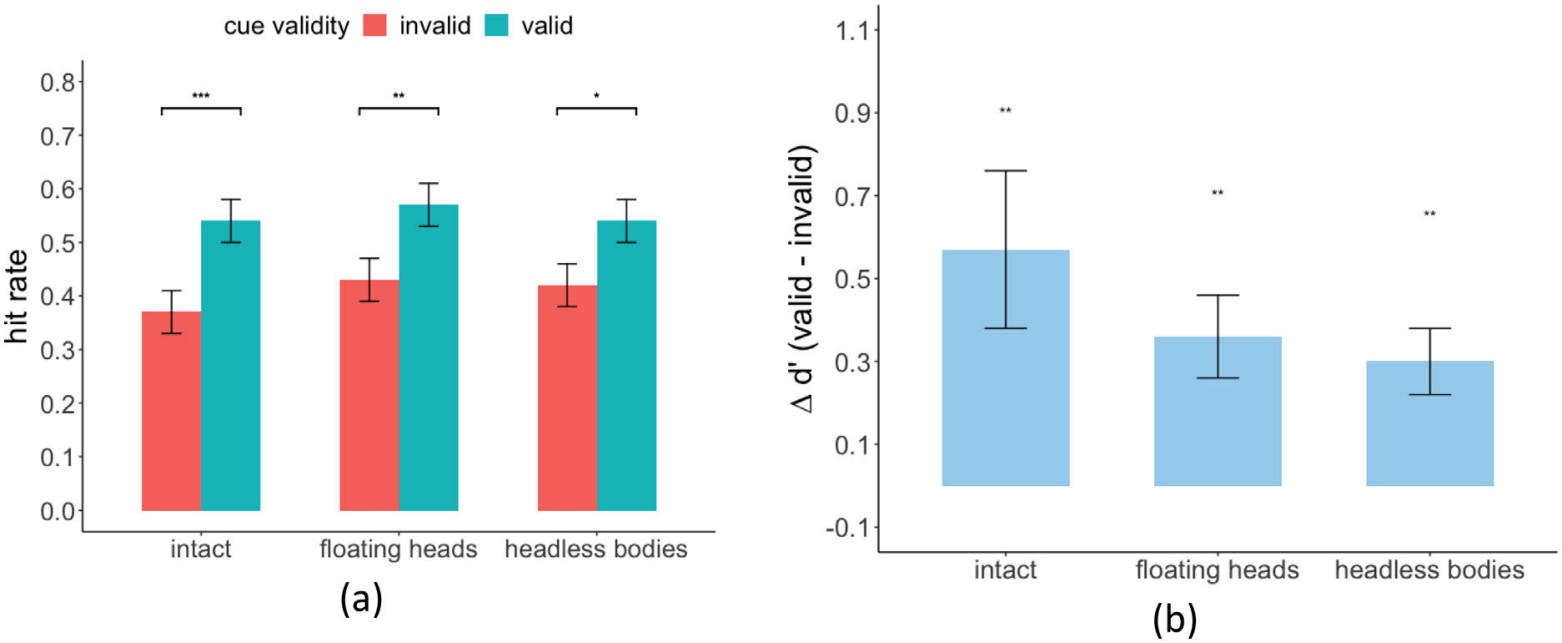
(**a**) Hit rate in three body parts/whole conditions separated by cue validity averaged across SOAs. (**b**) Sensitivity difference ∆d’ (valid d’ – invalid d’) in three body parts/whole conditions. All error bars represent standard errors across subjects.

We analyzed the effect of gaze cues on target detection performance. A three-way (body parts/whole condition × cue validity × delay) within-subject ANOVA test found that the hit rate for valid gaze cue trials was significantly higher than that for invalid cue trials in all three conditions (F(1, 19) = 26.24, *p* = 6.1e-05). There was no significant main effect of body parts/whole condition, F(2, 38) = 1.45, *p* = 0.25, or SOA, F(1, 19) = 0.523, *p* = 0.48. For simplicity, we reported the results averaged across the two SOAs (Figure 10a; for detailed results see Table 7). Two-way (body parts/whole condition × delay) within-subject ANOVA showed no significant main effect of SOA, F(1, 19) = 1.48, *p* = 0.24, and no significant main effect of body parts/whole condition on the false positive rates (FPR), F(2, 38) = 0.93, *p* = 0.41, intact FPR = 19.6%, SE = 3.3%, floating heads FPR = 18.8%, SE = 3.8%, and headless bodies FPR = 17.7, SE = 3.14%. We quantified the behavioral gaze cueing effect by computing an index of detectability, d’, for the valid and invalid gaze cue trials and taking their difference: ∆d’ = d’_valid_-d’_invalid_). Multiple paired *t*-test results showed that **∆**d’ in all three body parts/whole conditions were all significantly greater than 0, intact ∆d’ = 0.64, SE = 0.22, *p* = 0.004, floating heads ∆d’ = 0.36, SE = 0.13, *p* = 0.001, headless bodies ∆d’ = 0.32, SE = 0.09, *p* = 0.001, with *p* values corrected by FDR. However, there was no significant difference in ∆d’ across the three conditions (intact versus floating heads and headless bodies), F(2, 38) = 0.94, *p* = 0.40 (Figure 10b).

**Table 7.** The hit rate (with standard errors in parentheses and *p* values in bold), separated by cue validity and SOAs and body parts/whole condition.

	200 ms SOA		500 ms SOA	
	Invalid	Valid	Invalid	Valid
Intact	37.4%	54.0%	38.00%	53.7%
	(4.6%)	(4.3%)	(5.1%)	(5.4%)
	***p* = 3.2e-08**		***p* = 0.002**	
Floating heads	44.0%	57.5%	43.0%	55.2%
	(5.3%)	(4.8%)	(4.5%)	(4.5%)
	***p* = 5.13 = e-06**		***p* = 2.8e-04**	
Headless bodies	41.5%	57.4%	42.8%	49.8%
	(5.2%)	5.4%)	(4.3%)	(5.0%)
	***p* = 4.43e-05**		***p* = 0.003**	

All *p* values for Tukey post hoc tests were corrected by false discovery rate (FDR). BOLD *p* values were significant.

#### Relationship between behavioral performance and eye movements

**Figure 11.**
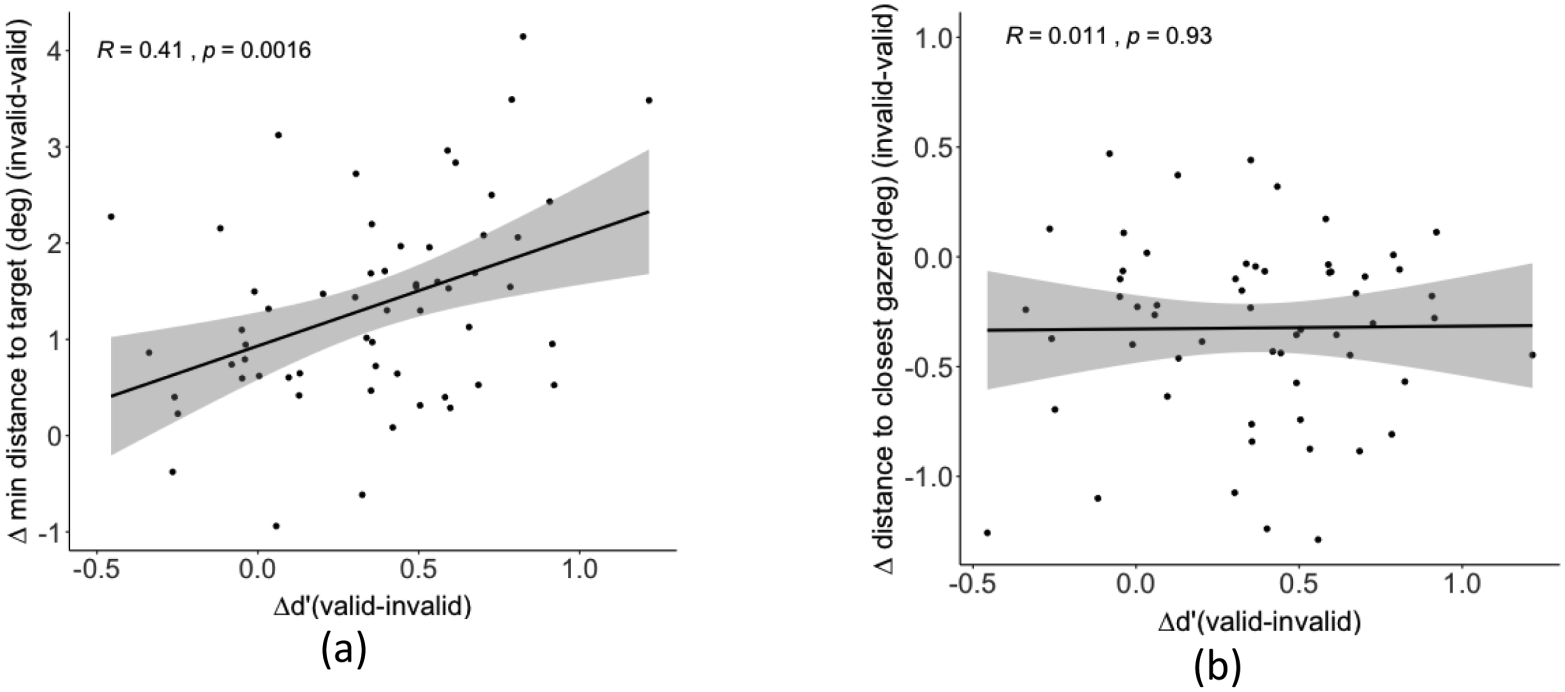
(**a**) Correlation between the ∆ minimum distance to the target (invalid – valid) to the target and ∆d’ (valid – invalid). (**b**) Correlation between ∆ distance to the closest gazer (invalid – valid) and ∆d’ (valid – invalid). Each dot represents a single participant in a condition (intact, floating heads, or headless bodies).

We investigated the relationship between the behavioral cueing effect measured by sensitivity (∆d’) and the eye movements cueing effect measured by fixation distance to the target (∆distance). We hypothesized that a larger influence of the gaze cue on the observers’ eye movement guidance toward the target would be related to higher gaze cueing effects on target detection accuracy. Indeed, we found a significant positive correlation r = 0.41, *p* = 0.0016, indicating that participants who showed a larger target detection difference between valid trails and invalid trials (∆d’ = d’ valid and d' invalid) also showed a larger difference in minimum distance to the target person (∆distance = distance invalid and distance valid; Figure 11a) for all conditions (two data points were identified as outliers and were excluded by correlation analysis). We also assessed the relationship between an observer's behavioral cueing effect and the observer's fixation distance to the gazers. Does an observer's tendency to look closer at gazers result in larger behavioral cueing effects? We found no significant relationship between the ∆fixation distance to the closest gazer and the behavioral cueing effect (∆d’), r = 0.011, *p* = 0.93 (Figure 11b).

## Discussion

Previous studies have mostly focused on assessing the influence of gaze on covert attention and eye movement with simple drawings or static images (Azarian et al., 2017; Bayliss et al., 2004a; Driver et al., 1999; Friesen & Kingstone, 1998; Hietanen, 2002; Kingstone et al., 2000). In this study, we investigated how movements of heads and bodies of dynamic gaze behaviors embedded in a rich visual environment influence eye movement in a visual search task. We presented natural videos where the gazers were at various eccentricities rather than at the fovea region. Our goal was to simulate a real-life scenario where there is dynamic gaze information and gazers often appear in the visual periphery. For our data set, the gaze cue was non-predictive of the target location to isolate exogenous influences from experiment-specific learned strategies (Droll, Abbey, & Eckstein, 2009; Druker & Anderson, 2010; Geng & Behrmann, 2005).

### The influence of gaze cue validity on eye movement search

We first analyzed how the fixation locations were affected by the gaze cues. Our studies showed an influence of gaze cue validity on eye movement guidance towards the target for the first three saccades. When the gazer/s looked toward the target (valid gaze cue trials) rather than to a distractor (invalid gaze cue trials), fixations were closer to the target, there were a larger proportion of trials with fixations within 2 degrees of the target, and a trend of shorter times for observers’ foveae to fall within 2 degrees of the target. A finer analysis of eye movement strategies showed that observers fixated (within 2 degrees) only on the gazers (and not the target) in a larger proportion of trials when the gaze cue was invalid. A likely explanation is that observers are following the invalid gaze cue and re-fixate the gazers when realizing that the target is not at the gaze goal.

### Contributions of head and lower body cueing to eye movement guidance

We investigated the separate contribution of the head and lower body by digitally deleting either the gazer's head or lower body, o neither (intact condition). We found a benefit of valid gaze cues in all three conditions but with the smallest effect for the headless bodies compared to the other two conditions in the first two fixations (see Figure 5d). Importantly, for the second fixations, the fixation distance to the target converged to a similar value for the intact and the floating heads condition, which was a distance than that second fixation to target distance in the headless bodies condition. These results suggest that head dynamics is the main source of guidance for eye movements. The lower body condition showed a small but significant cueing effect in guiding eye movements closer to the target. The results on eye movement guidance are complementary to a previous study showing the greater cueing influence of the head than the lower body on covert attention and microsaccades when observers maintain fixation during search (Han & Eckstein, 2022).

What might be the reason for the smallest gaze cueing effect for headless bodies? One possibility is that observers cannot extract reliable information about the gazed location from the lower body. To separately quantify the inherent information in the head and lower body that observers can extract to estimate gaze goals, we analyzed the explicit perceptual judgment of gaze direction on static frames from the videos. We showed that observers could accurately estimate gaze goals when the head was present but that estimation errors were large when only the lower body was present. This is consistent with previous results showing that head orientation plays a more important role in gaze perception (Florey, Clifford, Dakin, & Mareschal, 2015). Furthermore, we found that the location distribution of the perceptual judgments on intact images was least similar to the eye movement patterns in the headless bodies videos, and most consistent with those in the intact videos. This indicates that the lesser degree of eye movement guidance with headless bodies in the search task is mediated by observers’ difficulty in extracting gaze information from lower bodies. The gaze information from lower bodies was likely further reduced in the search task because the first saccade decisions are based on gazers appearing in the visual periphery (Loomis et al., 2008; Palanica & Itier, 2014; Yokoyama & Takeda, 2019).

#### Gaze cueing and behavioral search accuracy

Our study also showed that the behavioral performance in detecting the target person was improved with valid gaze cues regardless of the type of gaze information (both head and body, head only, or body only). This is consistent with a higher proportion of trials where participants only looked at the gazers and failed to foveate the target person when the gaze cue is invalid. In addition, a higher proportion of trials where participants foveated at the target when the cue was valid. Better behavioral performance with valid gaze cues was likely due to the guidance of eye movement toward the target. The benefit of valid gaze cues on behavioral performance showed no difference across different body parts/whole conditions. This might seem inconsistent with the previous study showing that headless bodies had the smallest cueing effect on the target detection performance (Han & Eckstein, 2022). However, in the Han and Eckstein (2022) study, observers maintained central fixation and were not allowed to execute eye movements. In the current study, we allowed free eye movements after the presentation of the gaze cues. Participants had enough eye movements to make eye movements closer to the target person likely reducing the differences across the three gaze cue conditions. If we had limited the display to two fixations or a shorter presentation time, we would likely obtain differences in target detection performance across body parts/whole conditions.

We also showed the correlation between the behavior performance in target detection and the fixation accuracy for all participants. A participant with a higher difference in their fixation location between valid and invalid cues tended to have a larger difference also in behavioral performance. This showed that the eye movement pattern was a strong indicator of perceptual decisions, which is consistent with previous studies on tasks such as visual search and face recognition (Chuk, Chan, & Hsiao, 2014; Eckstein, Beutter, Pham, Shimozaki, & Stone, 2007; Koehler & Eckstein, 2017b; Malcolm & Henderson, 2009; Peterson & Eckstein, 2012).

Table 8 provides a summary of all the main results in our study relating to the influence of gaze cue validity and the presence of the gazer's head.

**Table 8.** Results summary.

		Factors	
Measured variables		Gaze cue validity	Presence of gazer's head
	Perceptual judgment of gaze goal estimation with no time limit (Figure 7)	Not applicable	✓
	First and second fixation location (Figures 5a, b)	✓	✓
	Third fixation location (Figure 5d)	✓	✗
	Time to fixate on the target (Figure 6b)	✓	✓
	Foveation behavior (Table 4)	✓	✗
	Target detection performance with 2 seconds of presentation (Figure 10).	✓	✗

A check represents a significant effect of a factor on the measured variable, a cross represents no significant effect on the measured variable.

#### Limitations of the current study

There were some limitations to our study. First, the perceptual judgment task estimating gaze goals presented static frames extracted from the videos and allowed participants to free-view the images. The task did not incorporate the information on the movement dynamics of the head and lower body that was present in the videos. The approach for the perceptual judgment gaze estimation task might have underestimated the amount of gaze information in the actual headless body videos. Second, our study design presented the gazers first and the target/distractors after an SOA of 200 or 500 ms to better isolate the effect of the gazer cue. This design might overestimate the influence of the gazer on eye movements. In real-world scenarios, gazers, targets, and distractors are simultaneously present. Thus, the observers might rely less on gaze cues when sensory information about the target is simultaneously available. Future studies should include the presence of the target and distractors during the dynamic gaze behavior to assess how their presence modulates the gaze cue validity effects in the same way target detectability influences the synthetic cue effects (Eckstein et al., 2013; Shimozaki, Eckstein, & Abbey, 2003).

Third, previous studies have shown that body orientation introduces gaze perception bias when presented together with head orientation (Hietanen, 2002; Moors et al., 2015). In this study, we did not control the relative angle between the heads and bodies during filming. So, it is unknown whether a larger relative angle, or a larger relative motion difference during dynamic gaze behavior would show a larger cueing effect in guiding eye movements. One possible future direction is to explicitly manipulate the head and body motion to test how the integration of head and body motion affects eye movement planning.

Fourth, our study concentrated on the head/body movement while a large amount of literature focuses on the influences of the gazer's eyes (Driver et al., 1999; Friesen et al., 2004; Langton, Watt, & Bruce, 2000; Mansfield et al., 2003; Ristic, Friesen, & Kingstone, 2002). Our study was relevant to gazers, the target, and distractors situated at a distance from the observers. The mean angle subtended by the heads in our videos (1.39 degrees, STD = 0.29 degrees). Given that the average vertical distance of the head is about 0.24 m (Lee, Hwang Shin, & Istook, 2006), that would match the angle subtended by a real-sized head viewed at a distance of 9.9 m (STD = 1.7 m) in real life. The average vertical length of human eyes spans 2.4 cm (Bekerman, Gottlieb, & Vaiman, 2014). At a 9.9 m distance, the eyes subtend a mean angle of 0.139 degrees (vertically) providing degraded information about gaze goals compared to the head orientation. Future studies should investigate gazers at smaller distances from the observer and assess how dynamic gazers’ eye and head movements are integrated and their interactions similar to some studies using static images of the head and gaze (Balsdon & Clifford, 2018; Cline, 1967; Langton, 2000; Langton, Honeyman, & Tessler, 2004; Otsuka, 2014).

To summarize, our study extended the gaze cueing effect to search tasks in cluttered scenes and demonstrated the importance of head movements in guiding eye movements and improving target detection performance.
